# Investigation of Overrun-Processed Porous Hyaluronic Acid Carriers in Corneal Endothelial Tissue Engineering

**DOI:** 10.1371/journal.pone.0136067

**Published:** 2015-08-21

**Authors:** Jui-Yang Lai, Hsiao-Yun Cheng, David Hui-Kang Ma

**Affiliations:** 1 Institute of Biochemical and Biomedical Engineering, Chang Gung University, Taoyuan, Taiwan, 33302, Republic of China; 2 Biomedical Engineering Research Center, Chang Gung University, Taoyuan, Taiwan, 33302, Republic of China; 3 Molecular Medicine Research Center, Chang Gung University, Taoyuan, Taiwan, 33302, Republic of China; 4 Limbal Stem Cell Laboratory, Department of Ophthalmology, Chang Gung Memorial Hospital, Taoyuan, Taiwan, 33305, Republic of China; 5 Department of Chinese Medicine, Chang Gung University, Taoyuan, Taiwan, 33302, Republic of China; Osaka University, JAPAN

## Abstract

Hyaluronic acid (HA) is a linear polysaccharide naturally found in the eye and therefore is one of the most promising biomaterials for corneal endothelial regenerative medicine. This study reports, for the first time, the development of overrun-processed porous HA hydrogels for corneal endothelial cell (CEC) sheet transplantation and tissue engineering applications. The hydrogel carriers were characterized to examine their structures and functions. Evaluations of carbodiimide cross-linked air-dried and freeze-dried HA samples were conducted simultaneously for comparison. The results indicated that during the fabrication of freeze-dried HA discs, a technique of introducing gas bubbles in the aqueous biopolymer solutions can be used to enlarge pore structure and prevent dense surface skin formation. Among all the groups studied, the overrun-processed porous HA carriers show the greatest biological stability, the highest freezable water content and glucose permeability, and the minimized adverse effects on ionic pump function of rabbit CECs. After transfer and attachment of bioengineered CEC sheets to the overrun-processed HA hydrogel carriers, the therapeutic efficacy of cell/biopolymer constructs was tested using a rabbit model with corneal endothelial dysfunction. Clinical observations including slit-lamp biomicroscopy, specular microscopy, and corneal thickness measurements showed that the construct implants can regenerate corneal endothelium and restore corneal transparency at 4 weeks postoperatively. Our findings suggest that cell sheet transplantation using overrun-processed porous HA hydrogels offers a new way to reconstruct the posterior corneal surface and improve endothelial tissue function.

## Introduction

The cornea is a thin tissue layer that is responsible for the protection of intraocular structures and refraction of light [1]. The endothelium lining the posterior corneal surface acts as a physiological barrier to maintain tissue homeostasis and plays an important role in modulating tissue clarity [2]. Due to donor shortage, corneal endothelial tissue engineering is a promising alternative to corneal transplantation [3]. As early as 1990, Insler et al. have reported a method to transplant corneal endothelial cells (CECs) expanded ex vivo on the carriers made of collagen-coated dextran [4]. In recent years, naturally occurring biopolymer such as chitosan has also been widely used to engineer CEC culture substrates. Liang et al. have demonstrated that a carrier membrane made of hydroxyethyl chitosan, gelatin, and chondroitin sulfate is suitable for CECs to attach and grow [5]. Later, Ozcelik et al. have shown that an ultrathin chitosan-poly(ethylene glycol) hydrogel film displays excellent characteristics (i.e., desirable mechanical, optical and degradation properties) for corneal endothelial tissue engineering applications [6]. However, it should be noted that the problems involving the implantation of CEC-populated carrier substrates may include poor graft-host integration, optical interference, risk of foreign body reaction, and disturbance of physiological function.

To avoid the presence of carrier materials between the implanted cells and the host tissue associated with traditional tissue engineering concepts, our group has presented an alternative strategy of bioengineering of human corneal endothelium for tissue reconstruction [7–10]. The structure and function of CEC sheets fabricated on thermo-responsive culture surfaces resemble those of native endothelial monolayer. However, the use of gelatin-based delivery carriers for CEC sheet transplantation may pose a risk of physiological adverse effects. The interrupted nutrient transport and unbalanced intraocular pressure occurs after intracameral implantation of gelatin discs with dense structure. A porous hydrogel carrier is therefore designed to improve the aqueous humor circulation and cell delivery performance. In vivo biocompatibility studies indicate that the enlarged pore structure of gelatin implants attributed to the gas bubbles and ice crystals in the manufacturing process can prevent host corneal ulceration and neovascularization [11]. Our data also demonstrate that the gas bubbles produced by a simple stirring operation significantly affect the pore size and distribution of freeze-dried carriers, thereby determining ocular tissue responses to material implants.

Although these findings are encouraging for further development of stirring-processed and freeze-dried gelatin carriers, the material itself may not be the best suitable for clinical practice. Gelatin is a denatured protein derived from animal collagen by partial acidic or alkaline hydrolysis [12]. Therefore, the application of gelatin-based cell sheet delivery system may raise the concern of bovine spongiform encephalopathy. In contrast, hyaluronic acid (HA) is a biopolymer naturally found in the eye including the aqueous humor and vitreous body. It is a linear anionic polysaccharide composed of long chains of repeating disaccharide units of d-glucuronic acid and *N*-acetyl-d-glucosamine. Because of their innocuous nature and high capacity for lubrication, water sorption and water retention, HA-related commercial products such as Healon and Viscoat have been used in reducing CEC damage during cataract surgery [13]. In view of this, it seems reasonable to apply the HA to construct ocular cell delivery systems. Nevertheless, the HA hydrogel carriers exhibit rapid dissolution in aqueous environments and are mechanically too weak to provide sufficient support for cell sheet delivery. We have previously investigated the effects of cross-linker type on chemical modification of HA and have found that as compared to glutaraldehyde, the carbodiimide is a safer agent to build molecular bridges to reinforce the structure of polysaccharide materials [14,15]. In order to improve the biological stability, the use of 1-ethyl-3-(3-dimethyl aminopropyl) carbodiimide (EDC) is considered to be helpful for the fabrication of cross-linked porous HA hydrogels as carriers in intraocular grafting of bioengineered CEC sheets.

Given that HA is highly viscous, the stirring operation for introducing gas bubbles in the gelatin solution is unable to produce similar effects. As reported in the literature, the overrun process can be used to entrap the injected air that further creates cavities within the bulk of the poly(vinyl alcohol) hydrogels [16] and gelatin scaffolds [17]. Therefore, this study aims to explore the feasibility of combining nitrogen gas injection with ice crystal formation in developing novel HA carriers with enlarged pore structure. The aqueous biopolymer solutions were injected with nitrogen and frozen at -20°C, followed by lyophilization. Subsequently, the overrun-processed and freeze-dried HA discs were cross-linked with EDC and characterized to investigate the factors related to the application potential of hydrogel carriers in corneal endothelial tissue engineering. The material samples were evaluated by determinations of their pore size, porosity, biodegradability, freezable water content, and glucose permeability. The ionic pump function of CEC cultures exposed to the carrier discs was examined by analyzing the Na^+^,K^+^-ATPase alpha 1 subunit (ATP1A1) expressions at gene and protein levels. In addition, the in vitro biocompatibility was determined using the live/dead assays. After detachment from thermo-responsive culture surfaces, the bioengineered CEC sheets were transferred by using cross-linked porous HA carriers prepared via overrun process. The cell/biopolymer constructs were confirmed by histological examination. A rabbit model with corneal endothelial dysfunction was used to evaluate the therapeutic efficacy of HA carrier/CEC sheet constructs. The eyes denuded of the corneal endothelium or traumatized corneas received only carrier implants served as controls. Clinical observations including slit-lamp biomicroscopy, specular microscopy, and corneal thickness measurements were performed to determine the postoperative outcomes of these animals. For the first time, the present study reports the development of overrun-processed porous HA hydrogels as cell sheet delivery carriers for corneal endothelial transplantation and reconstruction.

## Materials and Methods

### Ethics Statement

The animal use protocols (CGU11-055) were reviewed and approved by the Institutional Animal Care and Use Committee of Chang Gung University.

### Materials

Hyaluronic acid sodium salt was obtained from Kewpie (Tokyo, Japan) as a dry powder. It was made by fermentation method and was highly purified. According to information from the supplier, the HA samples used in this study had a weight-average molecular weight of around 1100 kDa. 1-ethyl-3-(3-dimethyl aminopropyl) carbodiimide hydrochloride, hyaluronidase type V from sheep testes (1770 units/mg), glucose, and glucose assay kit (glucose oxidase/peroxidase reagent and o-dianisidine reagent) were purchased from Sigma-Aldrich (St. Louis, MO, USA). Balanced salt solution (BSS, pH 7.4) was obtained from Alcon Laboratories (Fort Worth, TX, USA). Phosphate-buffered saline (PBS, pH 7.4) was purchased from Biochrom AG (Berlin, Germany). Medium 199, gentamicin, Hanks’ balanced salt solution (HBSS, pH 7.4), trypsin-ethylenediaminetetraacetic acid (EDTA), and TRIzol reagent were purchased from Gibco-BRL (Grand Island, NY, USA). Collagenase type II was purchased from Worthington Biochemical (Lakewood, NJ, USA). Fetal bovine serum (FBS) and the antibiotic/antimycotic (A/A) solution (10000 U/ml of penicillin, 10 mg/ml of streptomycin, and 25 μg/ml of amphotericin B) were obtained from Biological Industries (Kibbutz Beit Haemek, Israel). All the other chemicals were of reagent grade and used as received without further purification.

### Preparation of Porous HA Carriers

The aqueous HA solutions of 0.5 wt% were prepared by dissolution of HA powder in double-distilled water. Before lyophilization at -55°C for 2 days, the aqueous solution was injected with nitrogen (gas flow rate = 250 sccm) for 10 min to generate gas bubbles and frozen at -20°C for 24 h to form ice crystals. Using the overrun process and freeze-drying method, the resultant samples were denoted as OFHA. Without the introduction of nitrogen, the prepared HA solution was subjected to freezing at -20°C for 24 h and lyophilized at -55°C for 2 days (FHA group). On the other hand, the aqueous HA solution was poured into a polystyrene planar mold (5 × 5 cm^2^, 1.5 cm depth) and air-dried at 25°C for 5 days to obtain the samples (designated as AHA) for comparison.

All fabricated HA hydrogel sheets were further cross-linked by immersing in an acetone/water mixture (85:15, v/v, pH 4.75) of 10 mM EDC [18]. The cross-linking reaction was allowed to proceed at 25°C for 2 days. The samples were thoroughly washed with double-distilled water to remove excess EDC and urea by-product. Using a 7-mm-diameter corneal trephine device, the hydrogel sheets were cut out to create carrier discs. The thicknesses of the material samples could be obtained by measuring at three different points with a Pocket Leptoskop electronic thickness gauge (Karl Deutsch, Germany), and the average was taken. Results were the average of five independent measurements. In this study, the sample thickness in the AHA, FHA, and OFHA groups was 714.2 ± 30.7, 693.4 ± 18.1, and 696.0 ± 25.8 μm, respectively. No significant difference was observed in the thickness value between these three groups (*P* > 0.05).

### Characterization of Porous Structure

Specimens were prepared for scanning electron microscopy (SEM) as described previously [19]. Small pieces of the hydrogel discs were cut off and mounted onto stubs using double-sided adhesive tape, and then gold coated in a sputter coater (Hitachi, Tokyo, Japan). The cross-section and surface morphologies of the HA carriers were examined using a Hitachi S-3000N SEM with an accelerating voltage of 10 kV. Twenty different pores were randomly selected, and the average pore diameters were calculated. Results were averaged on four independent runs.

The solvent replacement method was used for porosity measurements [20]. Each HA disc was first dried to constant weight (*W*_i_) in vacuo. The test samples were immersed in absolute ethanol overnight, blotted with tissue paper to remove excess ethanol on the surface, and weighed (*W*_f_) immediately. The porosity (%) was calculated as ((*W*_f_-*W*_i_)/*Vρ*) × 100, where *V* is the volume of the hydrogel disc and *ρ* is the density of absolute ethanol. Results were averaged on four independent runs.

### In Vitro Degradation Tests

To measure the extent of degradation, each test HA disc was first dried to constant weight (*W*_i_) in vacuo and was immersed in BSS containing 400 units/ml hyaluronidase at 34°C (physiological temperature of the cornea) with reciprocal shaking (50 rpm) in a thermostatically controlled water bath. Degradation medium was replaced weekly with fresh buffer solution containing the same concentration of enzyme [21]. At specific time intervals, the samples were taken out and washed with double-distilled water. The degraded samples were further dried in vacuo and weighed to determine the dry weight (*W*_d_). The in vitro degradability (%) was calculated as ((*W*_i_-*W*_d_)/*W*_i_) × 100. Results were the average of five independent measurements.

### Determination of Freezable Water Content

Differential scanning calorimetry (DSC) measurements were used to examine the states of water in the HA carriers. The disc samples were placed in a DSC cell (TA Instruments, New Castle, DE, USA), cooled to -20°C to freeze the swollen hydrogels, and heated to 20°C at a heating rate of 5°C/min under a nitrogen gas flow. The amount of freezable water was evaluated from the DSC endothermic ice-melting profile of the frozen hydrogel [22]. The enthalpy of melting (Δ*H*_m_) obtained by integration and normalization is in unit of J/g of swollen hydrogel. Temperatures and enthalpies of melting of the samples were calibrated using pure water as the standard. The latent heat of water is 333.5 J/g of pure water. The gram of freezable water per gram of swollen HA hydrogel (*W*_fH_/*W*_s_) was calculated as Δ*H*_m_/333.5. Results were the average of five independent measurements.

### Glucose Permeation Studies

Glucose permeation studies were performed at 34°C using a horizontal glass diffusion cell (PermeGear, Hellertown, PA, USA) having two stirred chambers with sampling ports [23]. The donor chamber was filled with a 6.9 μmol/ml (the glucose concentration of aqueous humor in rabbit) glucose solution in BSS (3 ml) and receptor chamber with BSS (3 ml). After immersion in BSS until fully swollen, the HA samples were placed between the two chambers. During the measurements, all solutions were stirred continuously to provide uniform solute distribution and to reduce boundary layering of glucose. After 8 h, the receptor chamber was sampled and analyzed using a glucose assay kit following the manufacturer’s instructions. Photometric readings at 540 nm were measured with a spectrophotometer (Thermo Scientific, Waltham, MA, USA) and compared with a standard curve of known glucose concentrations. Results were averaged on six independent runs.

### Rabbit Corneal Endothelial Cell Cultures

All animal procedures were performed in accordance with the ARVO Statement for the Use of Animals in Ophthalmic and Vision Research. Twenty adult New Zealand white rabbits (National Laboratory Animal Breeding and Research Center, Taipei, Taiwan, ROC) were used for in vitro biocompatibility studies. Primary rabbit CECs were prepared according to previously published methods [24]. Briefly, the animals were anesthetized intramuscularly with 2.5 mg/kg body weight of tiletamine hydrochloride/zolazepam hydrochloride mixture (Zoletil; Virbac, Carros, France) and 1 mg/kg body weight of xylazine hydrochloride (Rompun; Bayer, Leverkusen, Germany) and were euthanized with CO_2_ gas. The corneas were excised after sacrifice. Under a dissecting microscope (Leica, Wetzlar, Germany), Descemet’s membrane with the attached endothelium was aseptically stripped from the stroma and washed three times with PBS. The Descemet’s membrane-corneal endothelium complex was digested using 2 mg/ml collagenase in HBSS for 1 h at 37°C. Thereafter, the CECs were collected and resuspended in regular culture medium containing Medium 199 as a basal medium, 10% FBS, 50 μg/ml gentamicin and 1% A/A solution. Cultures were incubated in a humidified atmosphere of 5% CO_2_ at 37°C. Medium was changed every other day. Confluent monolayers were subcultured by treating with trypsin-EDTA for 2 min and seeded at a 1:4 split ratio. Only second-passage cells were used.

### In Vitro Biocompatibility Studies

Rabbit CECs (7 × 10^4^ cells/well) were seeded in 24-well plates containing regular growth medium and incubated overnight to allow cell attachment. After 1 week of cultivation, the HA discs sterilized in a graded series of ethanol solutions were placed on the apical cell surface in direct contact with the confluent cultures. Rabbit CEC cultures without contacting disc samples served as control groups. Following incubation for 8 h, the cell viability was determined using the Live/Dead Viability/Cytotoxicity Kit from Molecular Probes (Eugene, OR, USA) [25]. This assay uses intracellular esterase activity to identify the living cells; the process cleaves the calcein acetoxymethyl to produce a green fluorescence. Ethidium homodimer-1 can easily pass through the damaged cell membranes of dead cells to bind to the nucleic acids, yielding a red fluorescence. After washing three times with PBS, the cultures were stained with a working solution consisting of 2 μl of ethidium homodimer-1, 1 ml of PBS, and 0.5 μl of calcein acetoxymethyl. Cells were then observed and imaged under fluorescence microscopy (Axiovert 200M; Carl Zeiss, Oberkochen, Germany).

On the other hand, the gene and protein expression levels were measured after 8 h of direct contact between the CEC cultures and sterilized HA carriers. The total RNA was isolated from cells with TRIzol reagent according to the manufacturer’s procedure [26]. Reverse transcription of the extracted RNA (1 μg) was performed using ImProm-II (Promega, Madison, WI, USA) and Oligo(dT)_15_ primers (Promega). The primers used to amplify the rabbit Na^+^,K^+^-ATPase alpha 1 subunit (ATP1A1) complementary DNA (cDNA) were 5’-GTCTTCCAGCAGGGCATGAA-3’ (sense) and 5’-TAAGGGCAACACCCATTCCA-3’ (antisense). The sequences of the primer pair used to amplify the internal control cDNA, glyceraldehyde-3-phosphate dehydrogenase (GAPDH), were 5’-TTGCCCTCAATGACCACTTTG-3’ (sense) and 5’-TTACTCCTTGGAGGCCATGTG-3’ (antisense). Quantitative real-time reverse transcription polymerase chain reaction (RT-PCR) was performed on a Light-Cycler instrument (Roche Diagnostics, Indianapolis, IN, USA) according to the manufacturer’s instructions with FastStart DNA Master SYBR Green I reagent (Roche Diagnostics). Each sample was determined in quadruplicate, and the gene expression results were normalized to the level of GAPDH mRNA.

For the preparation of protein extracts, cells from each group were lysed in 1% NP-40 lysis buffer containing 1 mM EDTA, 1 mM ethylene glycol tetraacetic acid, 5 μg/ml antipain, 5 μg/ml pepstatin A, 1 mM phenylmethylsulfonyl fluoride, and 5 μg/ml aprotinin [27]. Protein concentrations were determined by protein assay (Bio-Rad, Hercules, CA, USA) and 50 μg of protein per lane was separated by electrophoresis under reducing conditions in 10% polyacrylamide gel with sodium dodecyl sulfate (SDS-PAGE). For Western blotting, SDS-PAGE gels were transferred to poly(vinylidene difluoride) membranes that were blocked with 5% nonfat milk in tris-HCl-buffered saline containing 0.1% Tween-20 (TTBS) for 1 h at room temperature. The membranes were then incubated with mouse anti-Na^+^,K^+^-ATPase alpha 1 subunit (1:1000; Upstate Biotechnology, Lake Placid, NY, USA) primary antibodies with 5% nonfat milk in TTBS overnight at 4°C with gentle rocking. Next, blots were washed for three times with 0.1% TTBS solution and incubated with secondary antibodies conjugated to horseradish peroxidase (1:5000; Chemicon International, Temecula, CA, USA) with 5% nonfat milk in TTBS for 1 h at room temperature. The SuperSignal West Pico chemiluminescent substrate (Pierce, Rockford, IL, USA) was used for detecting a secondary antibody on imaging films (Biomax MS, Eastman Kodak, Rochester, NY, USA). Anti-alpha-tubulin (1:2000; Abcam, Cambridge, MA, USA) was used as loading controls.

### Fabrication of HA-CEC Sheet Constructs

Second-passage rabbit CECs were used for this study. As described previously, the bioengineered CEC sheets were harvested from the thermo-responsive culture surfaces by reducing the incubation temperature from 37°C to 20°C [9]. After cell sheet detachment from the PNIPAAm-grafted culture dishes, the sterilized hydrogel carriers from OFHA groups were immediately placed on the apical surface of cell layers to create HA-CEC sheet constructs. Then, the cell/biopolymer samples were examined by histological analysis. The constructs were mounted onto precooled chucks in embedding medium (OCT Tissue-Tek; Sakura Finetek, Torrance, CA, USA) and frozen at -70°C. The specimens were cut with the use of a cryostat into 5 μm sections at -20°C. After fixation with 4% paraformaldehyde, the sections were stained with Hoechst 33258 (Invitrogen, Carlsbad, CA, USA) and observed under a fluorescence microscope (Carl Zeiss) to visualize cell nuclei.

### In Vivo Transplantation Studies

Eighteen adult New Zealand white rabbits (National Laboratory Animal Breeding and Research Center) weighing 3.0–3.5 kg and the hydrogel carriers from OFHA groups sterilized in a graded series of ethanol solutions were used for in vivo transplantation studies. All animal procedures were approved by the Institutional Review Board and were carried out in accordance with the ARVO Statement for the Use of Animals in Ophthalmic and Vision Research. Surgery was performed in the single eye of animals, with the normal fellow eye. Due to its high regenerative capacity, rabbit corneal endothelium was treated with mitomycin-C (Sigma-Aldrich) to establish an animal model mimicking conditions of human corneas [10]. The rabbits were anesthetized intramuscularly with 2.5 mg/kg body weight of tiletamine hydrochloride/zolazepam hydrochloride mixture and 1 mg/kg body weight of xylazine hydrochloride, and topically with two drops of 0.5% proparacaine hydrochloride ophthalmic solution (Alcaine; Alcon-Couvreur, Puurs, Belgium). In the operated eye of each rabbit, 0.1 mg/ml of mitomycin-C was injected into the anterior chamber. After treatment for 2 weeks to prevent CEC proliferation and migration, the rabbits were anesthetized again under the same conditions as mentioned above. The cornea was penetrated near the limbus with a slit knife under the surgical microscope (Carl Zeiss), and the central 7 mm of corneal endothelium was scrapped gently with a silicone-tipped cannula without damaging the underneath Descemet’s membrane.

Once detached from the thermo-responsive culture surfaces, the rabbit CEC sheets were immediately attached to the carriers from OFHA groups to fabricate cell/biopolymer constructs, as described in the previous section (*Fabrication of HA-CEC sheet constructs*). After disinfection and sterile draping of the operation site, the pupil was dilated with one drop of 1% atropine sulfate (Oasis, Taipei, Taiwan, ROC), and a lid speculum was placed. Then, the constructs were implanted in the anterior chamber through a 7.5-mm corneal/limbal incision (OFHA+CEC group, *n* = 6). The incision site was closed with 10–0 nylon sutures. Traumatized rabbit corneas received OFHA carrier implants only (OFHA group, *n* = 6) or no transplantation (Wound group, *n* = 6) were the controls. After surgery, 1% chlortetracycline hydrochloride ophthalmic ointment (Union, Taipei, Taiwan, ROC) was immediately applied to the ocular surface in all three groups. Each surgical eye received two drops of 0.3% gentamicin sulfate (Oasis) and one drop of 1% prednisolone acetate (Allergan, Westport, Co. Mayo, Ireland) four times a day during the follow-up period of 4 weeks. Daily inspection was continued for the whole course of the experiment. All animals were housed in temperature (22°C) and humidity (45%) controlled rooms. They had access to food and water *ad libitum*. None of the rabbits showed signs of pain or distress during postoperative monitoring. At the end of the experiments, the animals were anesthetized intramuscularly with 2.5 mg/kg body weight of tiletamine hydrochloride/zolazepam hydrochloride mixture and 1 mg/kg body weight of xylazine hydrochloride and were euthanized with CO_2_ gas.

### Clinical Observations

Biomicroscopic examinations were carried out as described elsewhere [28]. The rabbits were anesthetized under the same conditions as for surgery. Ophthalmic evaluations were done after 4 weeks of transplantation. The corneal clarity was assessed using slit-lamp biomicroscopy (Topcon Optical, Tokyo, Japan). The CEC density in the rabbit eyes was measured by specular microscopy (Topcon Optical) [29]. Each data point was an average of three independent observations. Central corneal thickness (CCT) was determined using an ultrasonic pachymeter (DGH Technology, Exton, PA, USA) with a hand-held solid probe [30]. During the measurements, the probe tip of the pachymeter was held perpendicular on the central cornea. An average of ten readings was taken.

### Statistical Analyses

Results were expressed as mean ± standard deviation (SD). Comparative studies of means were performed using one-way analysis of variance (ANOVA). Significance was accepted with *P* < 0.05.

## Results and Discussion

### Preparation of Porous HA Carriers

The development of porous structure is one of the important issues in current research field of tissue engineering [31]. An accurate control of pore size and distribution of biomaterials is required to regulate nutrient uptake to and waste removal from the cultured cells. Porous carriers can be fabricated by a simple freeze-drying technique using water as porogen solvent [32]. We, as others, have adopted this method to create porosity in gelatin hydrogels [11,33,34]. In order to enlarge the pore structure of freeze-dried biopolymers, a simple stirring operation for introducing gas bubbles in the aqueous gelatin solution has also been proposed [11,20,22]. Herein, using HA as an alternative biomaterial for intraocular delivery of bioengineered CEC sheets, the cross-linked porous hydrogel discs were prepared via overrun process and freeze-drying method. To the best of our knowledge, the application potential of these cell carriers in corneal endothelial tissue engineering is yet to be investigated.

### Characterization of Porous Structure

**Fig 1:**
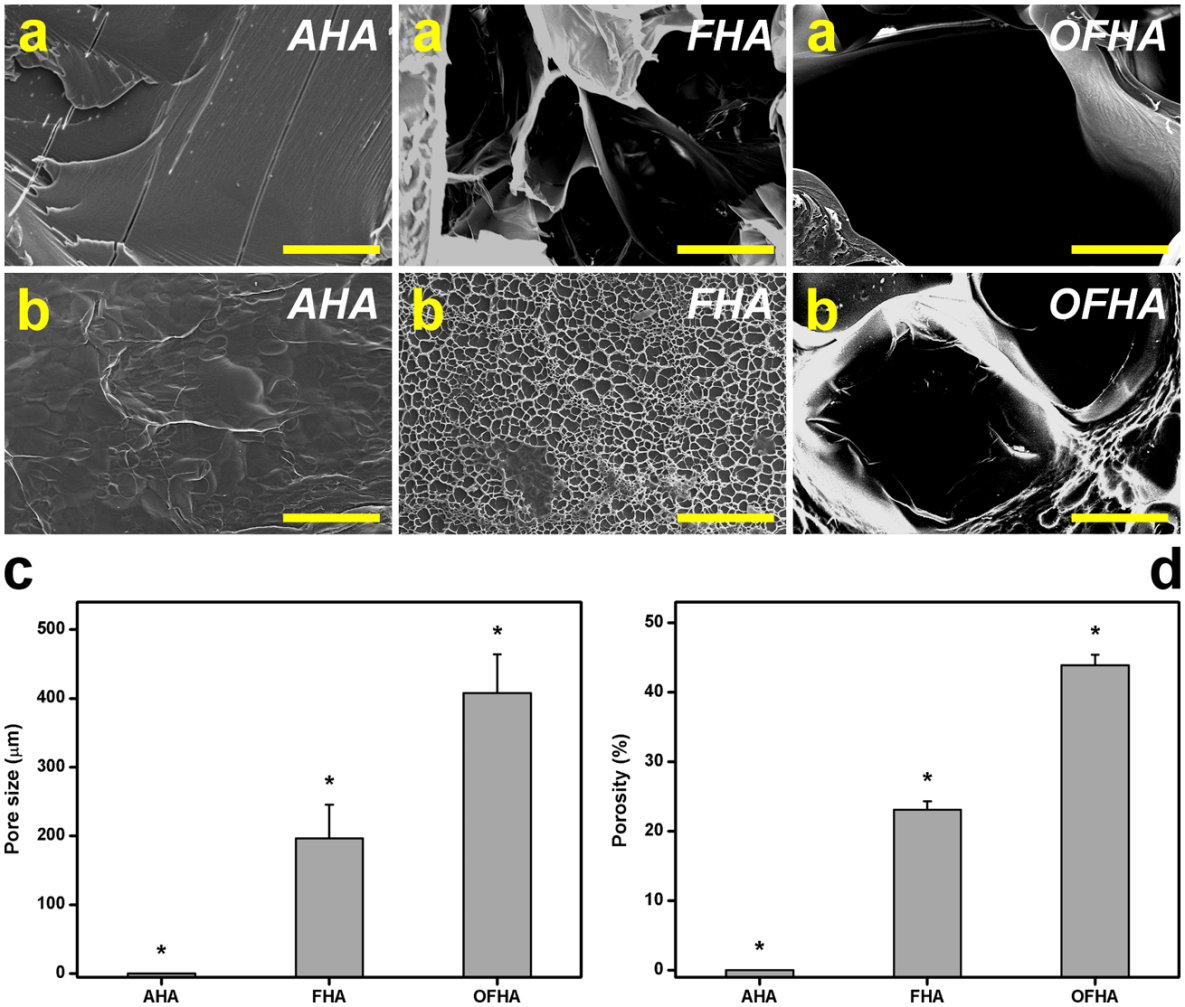
(a) Cross-section and (b) surface images obtained by scanning electron microscopy of the HA carriers. Scale bars: 100 μm. (c) Pore size and (d) porosity of various HA carriers. Values are mean ± SD (*n* = 4). *P < 0.05 vs all groups.

Fig 1a and 1b shows the cross-sectional and surface SEM images of the EDC cross-linked HA carriers, respectively. In the AHA groups, the samples had a dense structure. No pores were observed in either the surface or the bulk region. By contrast, the discs from the FHA and OFHA groups presented porous three-dimensional structure. Two different pore sizes were produced in the materials. The carriers prepared via overrun process and freeze-drying method contained larger pores in their interior. In addition, while a number of interconnected pores could be formed on the surface of OFHA samples, a dense surface skin layer was found in the discs from FHA. These findings support the observation that the cryogenic treatment of chemically cross-linked gelatin hydrogels causes skin formation during fabrication of porous biopolymer scaffolds [34]. However, the introduction of gas bubbles in the aqueous HA solutions can overcome this structural limitation to mass transfer given that the surface of obtained disc samples is porous. In the present work, the differences in carrier structure between air-dried, freeze-dried, and combined overrun process/freeze-drying groups are probably due to the influence of fabrication methods.

The results of further quantification of porous structure of EDC cross-linked HA carriers are shown in Fig 1c and 1d. Both the values of pore size and porosity of AHA samples were almost zero, indicating that the air-drying of aqueous solutions to form the solid does not create pores. By means of ice crystal formation, the freeze-dried disc samples from FHA groups exhibited the pore size and porosity of 197 ± 49 μm and 23.1 ± 1.2%, respectively. It is noteworthy that at the same processing conditions (i.e., freezing at -20°C for 24 h and lyophilization at -55°C for 2 days), the carbodiimide cross-linked gelatin hydrogels have an average porosity of 31.5% [11], which is significantly higher than their HA counterparts. One possible explanation is that the highly viscous HA solution decreases the ability to form large ice crystals around the polysaccharide molecules. In the OFHA groups, significantly higher pore size (408 ± 56 μm) and porosity (43.9 ± 1.5%) were observed than in the FHA groups (*P* < 0.05), suggesting that a further use of nitrogen gas injection can effectively create additional cavities within the bulk of the biopolymer hydrogel carriers.

### In Vitro Degradation Tests

**Fig 2:**
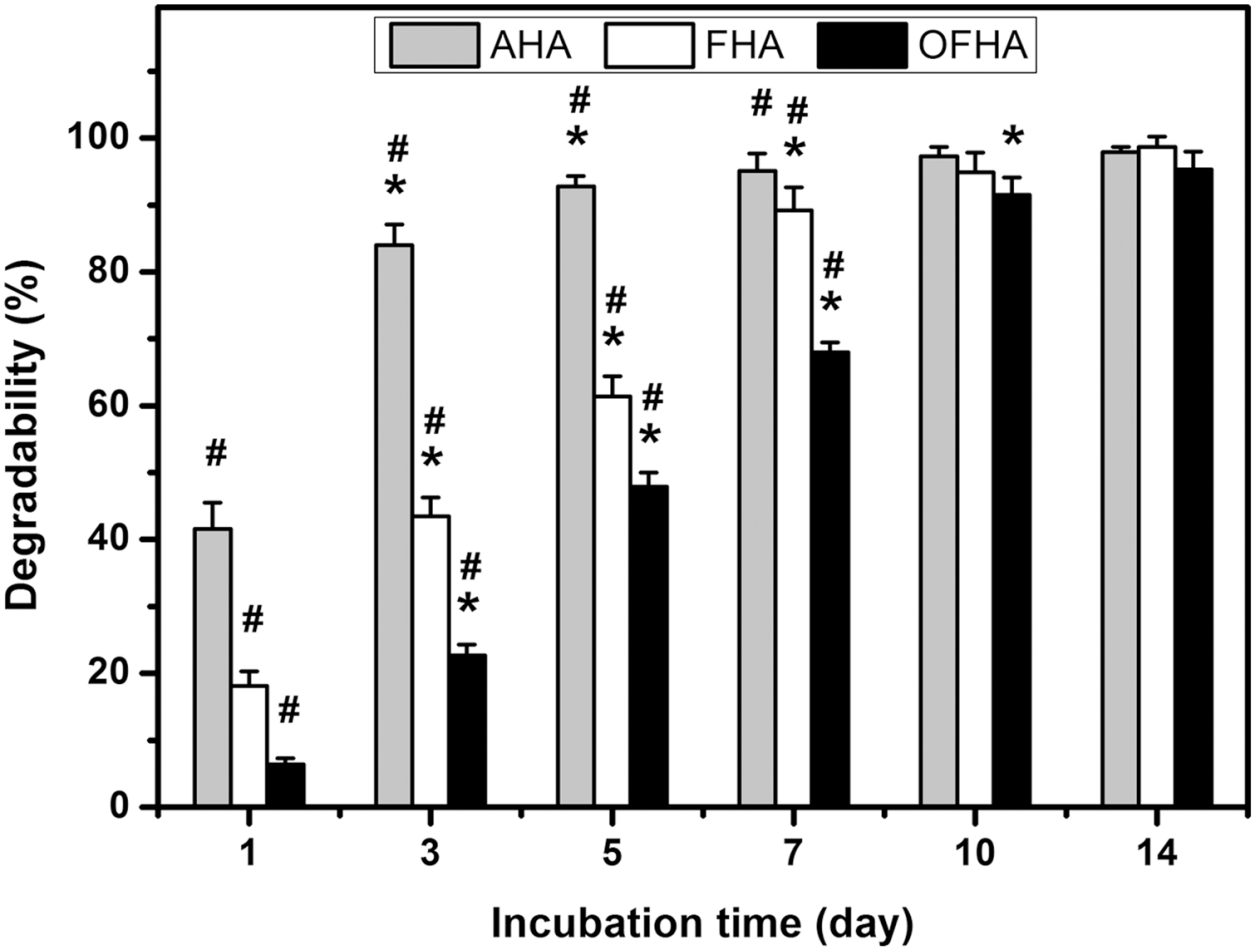
Time course of in vitro degradability of various HA carriers after incubation at 34°C in BSS containing hyaluronidase. An asterisk indicates statistically significant differences (*P < 0.05; *n* = 5) for the mean value of degradability compared with the value at the previous time point. ^#^ *P* < 0.05 vs all groups (compared only within each time point group).

The interest in using naturally occurring biopolymers for the fabrication of delivery carriers is their degradability under physiological conditions [12,35]. HA is a polysaccharide that can be degraded by endogenous hyaluronidase. In this study, the resistance against enzymatic degradation of cross-linked porous HA carriers was determined by incubation of disc samples in BSS containing hyaluronidase (Fig 2). After 1 day, the in vitro degradability in the AHA, FHA, and OFHA groups was 41.6 ± 3.9, 18.1 ± 2.2, and 6.4 ± 0.9%, respectively. The values showed significant differences between these three groups (*P* < 0.05). It is known that the biological stability is a crucial parameter to measure the extent of cross-linking of chemically modified hydrogels. After carbodiimide cross-linking, various ophthalmic biomaterials such as amniotic membrane [36], gelatin [37], and HA [14] have been evaluated by this methodology to quantify the cross-linking degree. Given that the cross-link formation can increase the resistance to enzymatic degradation, the overrun-processed and freeze-dried carrier samples have the greatest extent of cross-linking among all the groups studied. Our results indicate that the exposure of highly porous HA disc to EDC may lead to the increase in the collision frequency between biopolymer molecules and chemical cross-linkers, thereby providing enhanced stability and structure to the carrier materials. For each disc type, the residual polysaccharide hydrogels gradually degraded with time from 1 to 14 days. The in vitro degradability of AHA, FHA, or OFHA samples exceeds 95% after 14 days of incubation in the presence of hyaluronidase. These findings suggest that when used in the body, the delivery carriers can be almost completely degraded, without causing problems associated with the permanent presence of foreign materials.

### Determination of Freezable Water Content

**Fig 3:**
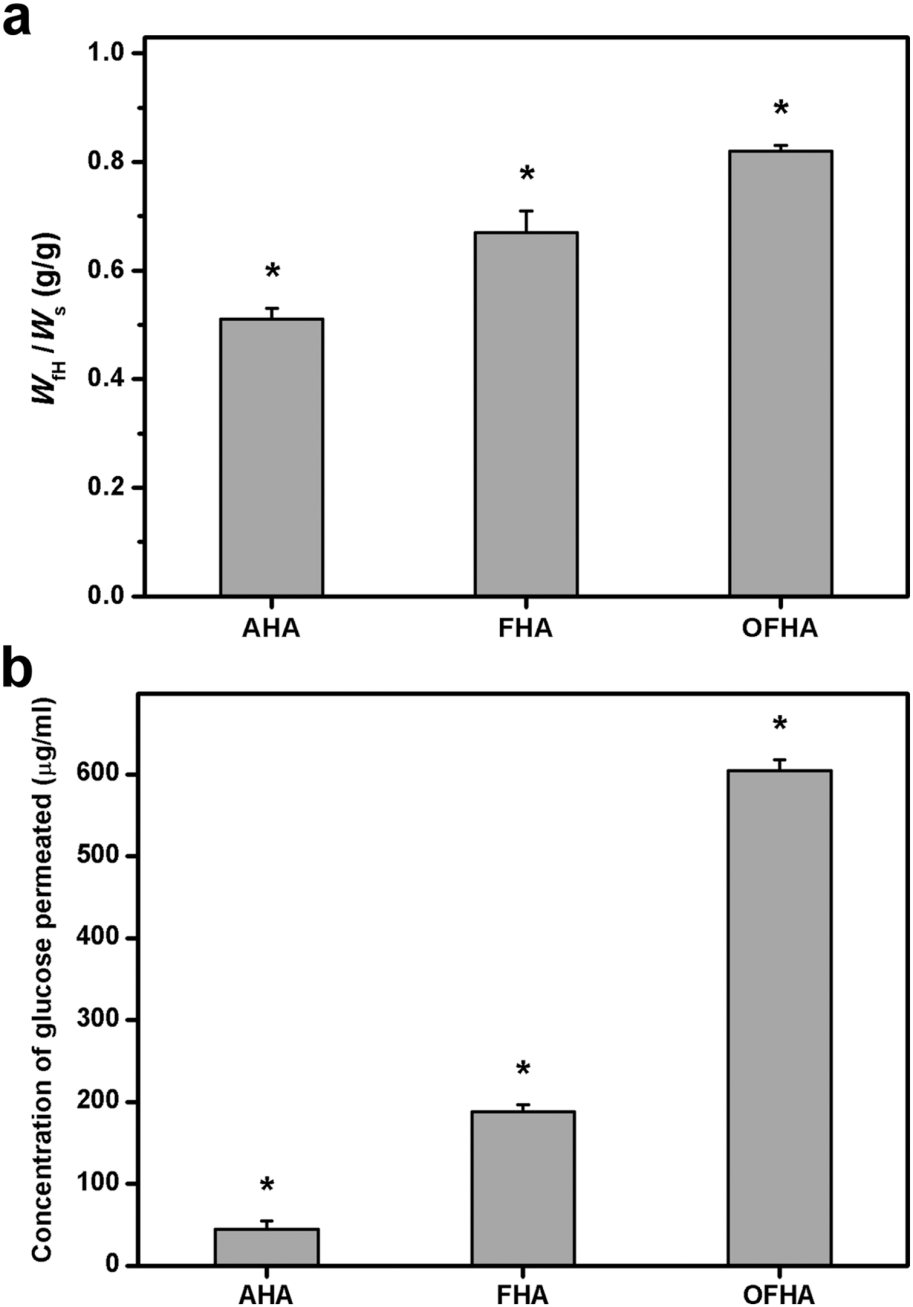
(a) Freezable water content (*W*_fH_/*W*_s_) of various HA carriers. Values are mean ± SD (*n* = 5). *P < 0.05 vs all groups. (b) Concentration of glucose permeated through various HA carriers at 34°C. Values are mean ± SD (*n* = 6). *P < 0.05 vs all groups.

It has been documented that the nature of water in hydrogel materials plays an important role in the solute diffusion mechanism [38]. In this study, the states of water in the HA hydrogels were determined by DSC measurements to investigate whether the cross-linked porous delivery carriers could have a higher fraction of water available for solute diffusion. The enhanced freezable water content is indicative of the elevated solute permeability of hydrogel carriers. As shown in Fig 3a, the total amount of freezable bound water and free water in swollen HA hydrogels from AHA, FHA, and OFHA groups was 0.51 ± 0.02, 0.67 ± 0.04, and 0.82 ± 0.01, respectively. The values showed significant differences between these three groups (*P* < 0.05). EDC is a chemical cross-linker that can induce ester bond formation between the carboxyl and hydroxyl groups of polysaccharide molecules [18]. Given that the hydrogen bonding exists between the water molecules and free carboxylic acid groups of HA, the extent of cross-linking of swollen biopolymer hydrogels may affect their freezable water content. This may explain that the highest mobile fraction of water is observed for the overrun-processed and freeze-dried carrier samples with the lowest amount of free carboxylic acid groups.

### Glucose Permeation Studies

Glucose permeability is one of most important prerequisites of carrier materials for CEC sheet delivery applications given that this nutrient is a principal source of energy for cornea. Fig 3b shows the concentration of glucose permeated through various HA hydrogel discs at 34°C. In the AHA groups, the detected glucose concentration was 44.6 ± 10.2 μg/ml, which was significantly lower than those in the FHA (187.6 ± 8.9 μg/ml) and OFHA (605.1 ± 13.4 μg/ml) groups (*P* < 0.05). These results indicate that the dense structure of biopolymer samples may restrict the permeation of nutrients. In addition, although the porous materials have lower resistance to glucose transport, the carriers prepared by using an additional nitrogen gas injection method can effectively increase the amount of permeated nutrients. We have previously shown that the enlarged pore structure of freeze-dried gelatin hydrogels created by a simple stirring operation is beneficial to glucose transport [20]. For the first time, the present data provide evidence that the elimination of problems related to dense surface skin formation may play a key role in the development of highly porous HA carriers for intraocular delivery of cell sheet grafts. It is also reasonable to assume that the residence of overrun-processed and freeze-dried polysaccharide implants in the anterior chamber will minimize the interruption of nutrient transport processes and disturbance of aqueous humor dynamics.

### In Vitro Biocompatibility Studies

**Fig 4:**
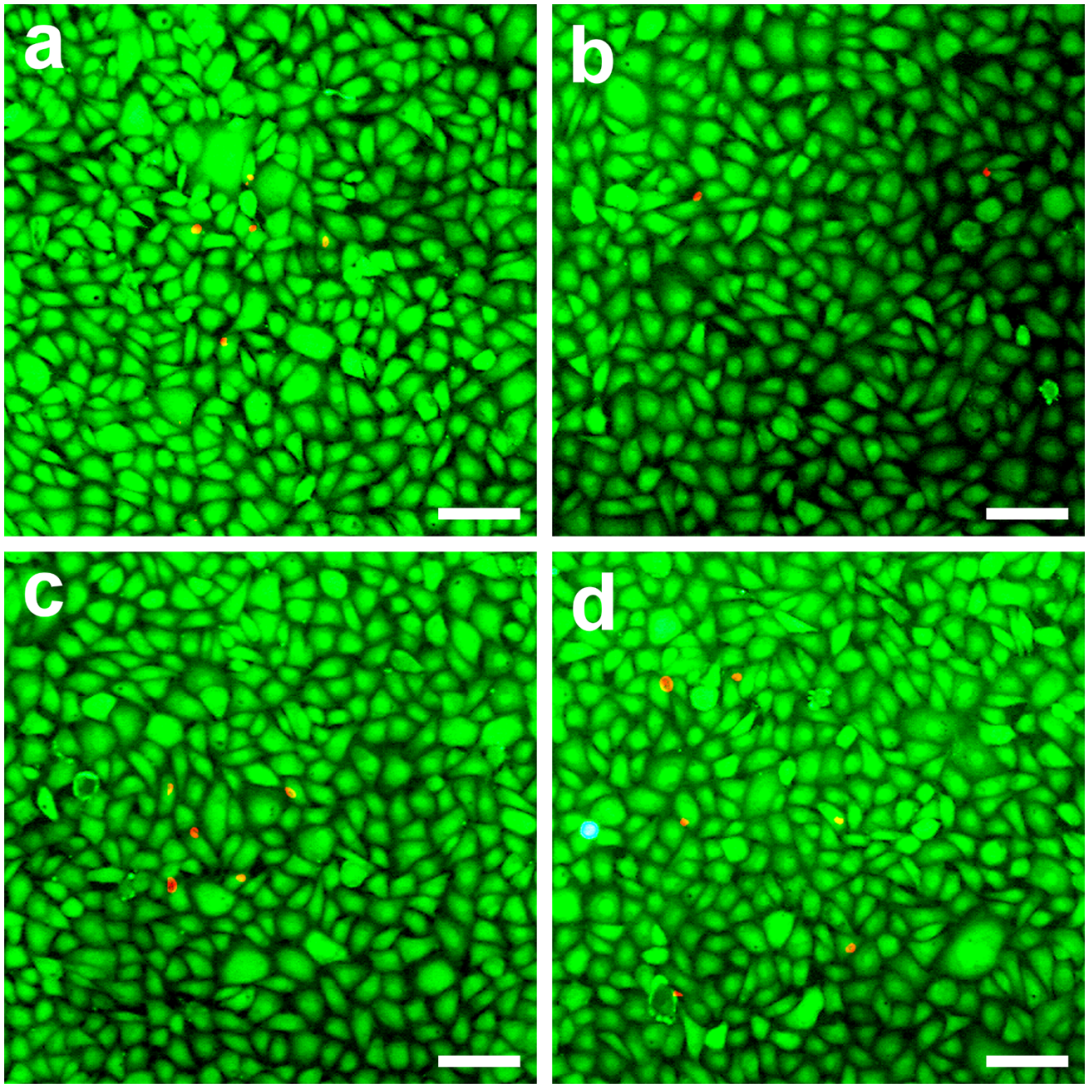
Cell viability of rabbit CEC cultures was determined by staining with Live/Dead Viability/Cytotoxicity Kit in which the live cells fluoresce green and dead cells fluoresce red. Fluorescence images of cells in (a) controls (without test materials) after 8 h of direct contact with different types of HA samples (b) AHA, (c) FHA, and (d) OHFA. Scale bars: 50 μm.

Fig 4 shows the results of live/dead staining to determine the viability of rabbit CECs exposed to various HA discs. In the control and all the experimental groups, the majority of cultures exhibit green fluorescence and only a few cells emit red fluorescence, indicating that irrespective of carrier structure, the biopolymer hydrogels have good compatibility with CECs. These observations are in accordance with our previous findings on the biocompatibility of cross-linked porous gelatin [22].

**Fig 5:**
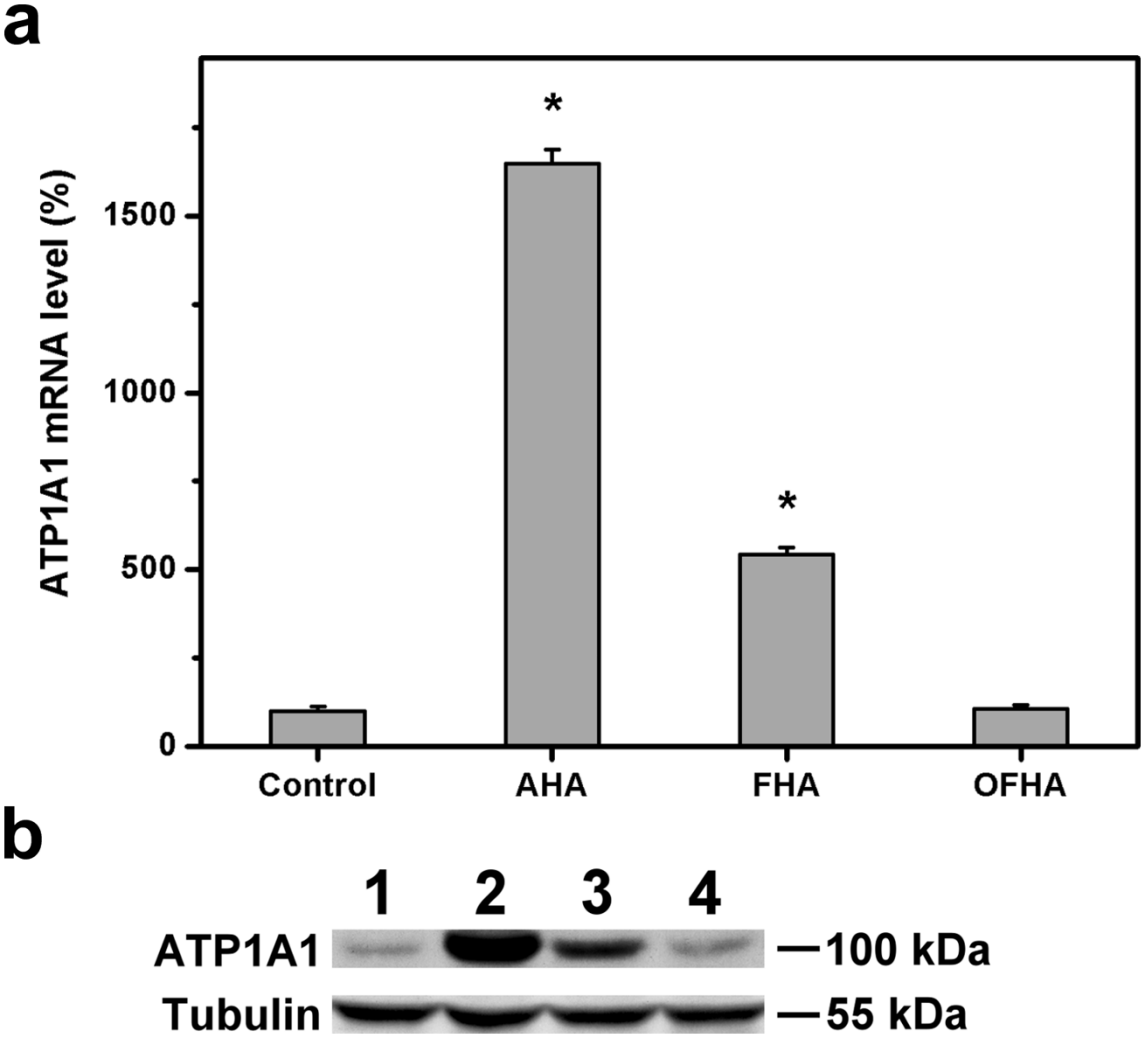
(a) Gene expression level of ATP1A1 in rabbit CECs after 8 h of direct contact with various HA carriers, measured by real-time reverse transcription polymerase chain reaction. Normalization was done by using GAPDH. Data in the experimental groups are percentages relative to that of control groups (cells cultured in the absence of HA materials). An asterisk indicates statistically significant differences (*P < 0.05; *n* = 4) as compared to the control groups. (b) Western blot analysis of ATP1A1 expression in the rabbit CECs after 8 h of direct contact with HA carriers. Lane 1: control (without HA materials), Lane 2: AHA, Lane 3: FHA, and Lane 4: OFHA groups.

The influence of carrier materials on corneal physiology was also investigated in vitro by measuring the ionic pump function of rabbit CECs. The gene expression of membrane transport protein such as Na^+^,K^+^-ATPase alpha 1 subunit (ATP1A1) was evaluated by using quantitative real-time RT-PCR. As shown in Fig 5a, the transcript level in the control groups was set to 100%. In the OFHA groups, the ATP1A1 gene expression was 106.6 ± 9.7%, which was not significantly different compared with control groups (*P* > 0.05), but was significantly lower than those of the AHA (1648.3 ± 40.5%) and FHA (542.9 ± 19.0%) groups (*P* < 0.05). To further correlate gene expression profile of ATP1A1 with protein, Western blotting was performed. As shown in Fig 5b, the CEC cultures from the control groups had a similar protein expression pattern to those of OFHA groups. Western blot analysis with a monoclonal antibody for ATP1A1 demonstrated a 100-kDa band in these cell lysates. By contrast, the bands were more intense in lanes 2 and 3. In particular, the protein signal was most pronounced from the CECs of the AHA groups. These findings suggest that the structure of HA hydrogel discs obtained from different fabrication techniques greatly affects the ionic pump function of rabbit CECs. While the samples with dense structure significantly up-regulate the ATP1A1 expression, the cross-linked porous materials improve the functional abnormality of this protein. It is noteworthy that without dense surface skin formation, the carriers have little or no effect on cellular homeostasis. The mechanism of altered expression of membrane transport protein caused by the decrease in CEC density has been elucidated [39]. As shown in Fig 4, the number of live cells with green fluorescence is reduced due to the direct contact of CEC cultures with discs from AHA groups, thereby drastically increasing the ATP1A1 expressions at gene and protein levels. The carriers prepared via overrun process and freeze-drying method are advantageous to use in bioengineered CEC sheet delivery as they do not produce cellular physiological responses.

### Fabrication of HA-CEC Sheet Constructs

**Fig 6:**
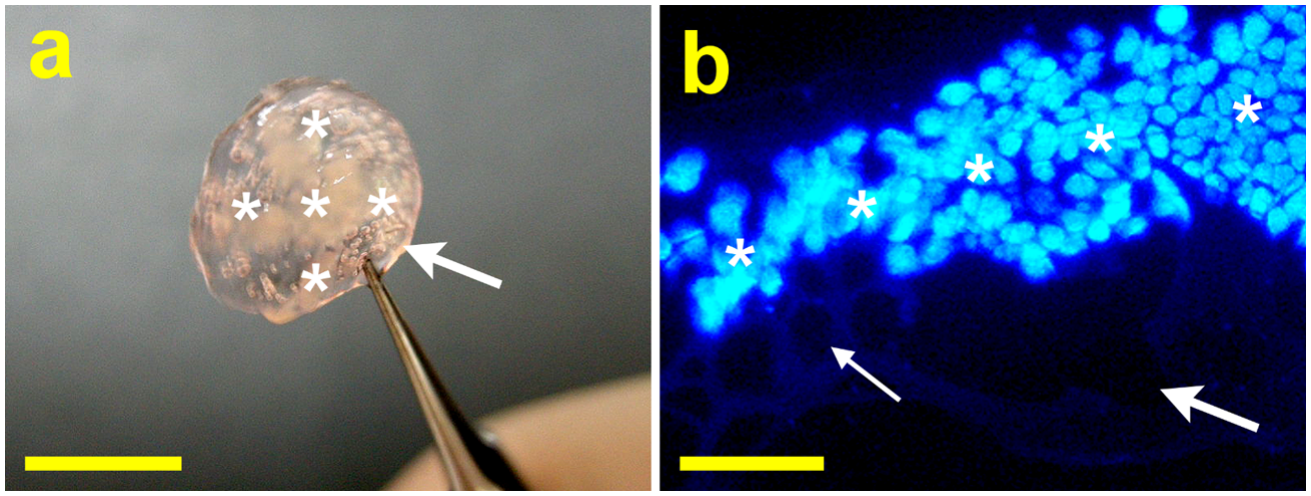
Fabrication of HA-CEC sheet constructs using thermo-responsive culture supports and porous delivery carriers. (a) Picture of bioengineered cell sheet graft (asterisk) attached to the porous hydrogel carrier (arrow) from OFHA group. Scale bars: 5 mm. (b) Light micrograph of cross-section of the construct stained with Hoechst 33258. Large arrow: larger pore on the interior of the carrier; Fine arrow: smaller pore on the surface of the carrier; Asterisk: cell sheet. Scale bars: 200 μm.

Recently, in our laboratory, HA has been used as an important extracellular matrix component for generating keratocyte culture microenvironment and controlling cell aggregation [40]. Because of their enhanced phenotypic maintenance and biosynthetic capacity, the bioengineered cell spheroids fabricated on HA coatings can be applied to corneal stromal tissue engineering. In this study, we also explore the feasibility of using cross-linked porous HA carriers for cell sheet transfer and corneal endothelial tissue engineering. Based on the aforementioned results, the hydrogel discs from OFHA groups show the greatest biological stability, the highest freezable water content and glucose permeability, and the minimized adverse effects on ionic pump function of CECs. Therefore, after detachment from the thermo-responsive culture surfaces, the bioengineered CEC sheets were transferred and attached to the overrun-processed and freeze-dried HA carriers to obtain cell/biopolymer constructs (Fig 6a). Under fluorescence microscopy, the cross-section stained with Hoechst 33258 was observed to examine the presence of cell sheets on the polysaccharide hydrogels. As shown in Fig 6b, the CECs positioned on the carriers with large interior pores emitted blue fluorescence. In addition, the well organization of cell monolayer was noted on the OFHA disc surfaces with small pores.

### In Vivo Transplantation Studies

**Fig 7:**
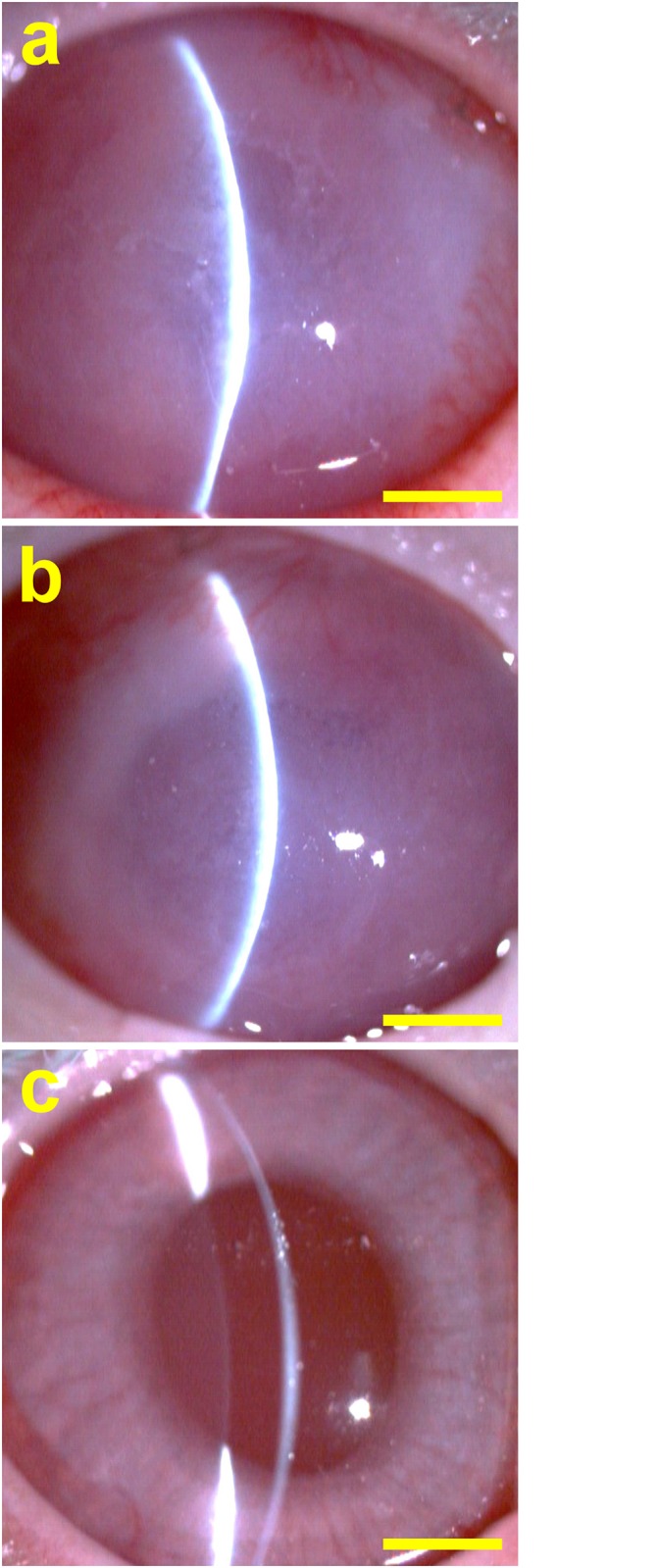
Representative slit-lamp biomicroscopic images of rabbit eyes 4 weeks after surgical treatment of corneal endothelial dysfunction. (a) Wound group: cornea denuded of endothelium, (b) OFHA group: endothelial scrape-wounded cornea implanted with HA carriers, and (c) OFHA+CEC group: endothelial scrape-wounded cornea implanted with HA carriers and bioengineered CEC sheet. Scale bars: 5 mm.

Due to its best suitability for CEC sheet delivery applications, the OFHA hydrogel discs are used to fabricate cell/biopolymer constructs for in vivo studies. Clinical observations are made to assist in understanding the therapeutic efficacy of construct implants in an animal model of corneal endothelial dysfunction. Fig 7 shows representative slit-lamp biomicroscopic images for each group. After surgery for 4 weeks, the rabbit corneas from the Wound groups were opaque (iris not visible). In addition, severe corneal swelling and cloudiness were noted as a result of mechanical stripping of native corneal endothelium from Descemet’s membrane. In the OFHA groups, the endothelial scrape-wounded corneas receiving biopolymer carriers presented similar opacities to those seen in the Wound groups. By contrast, the corneas in the rabbits from OFHA+CEC groups were clear, indicating that the bioengineered cell sheets delivered by hydrogel carriers can successfully reconstruct the corneal posterior surface.

**Fig 8:**
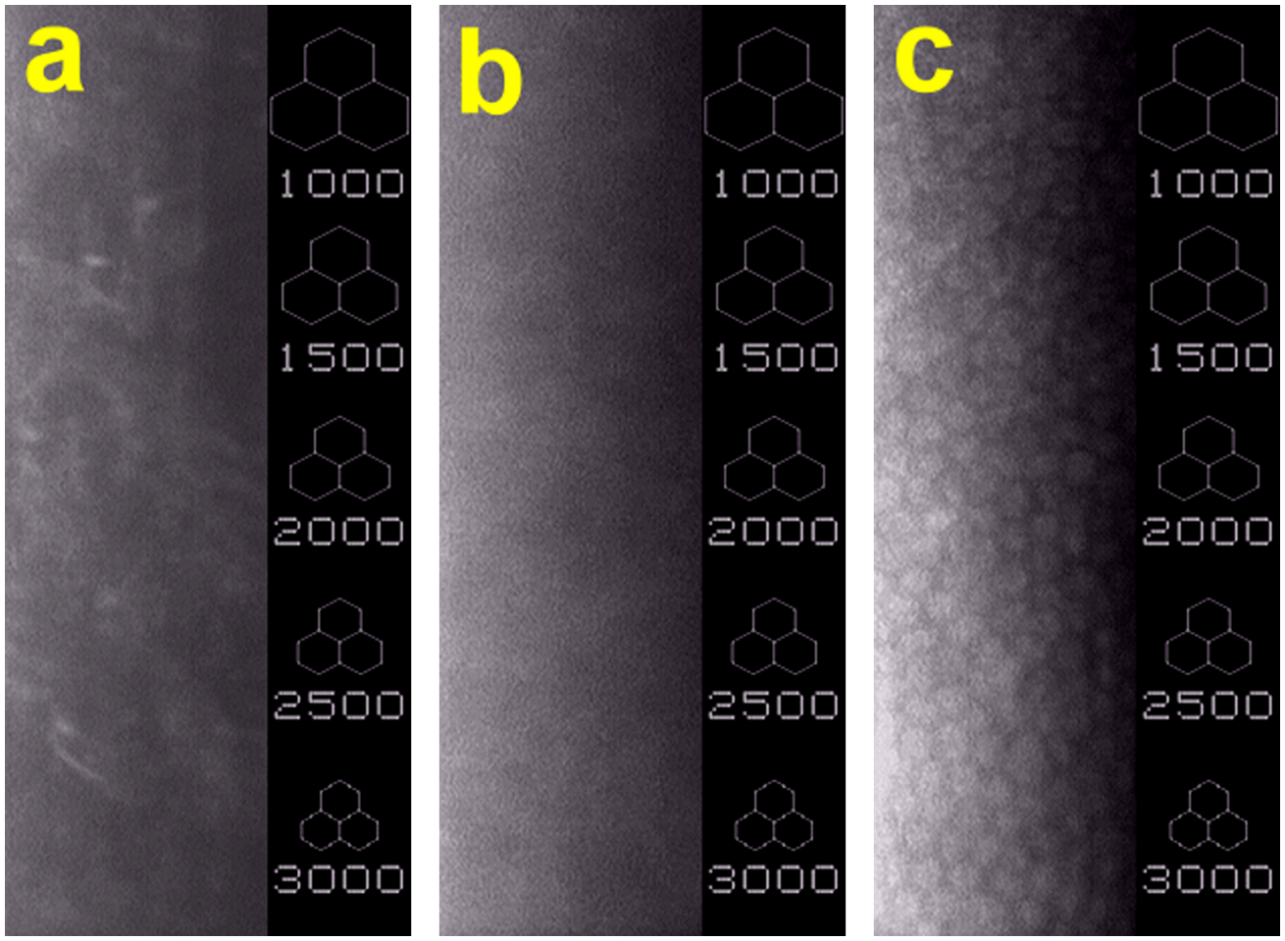
Representative specular microscopic images of rabbit eyes 4 weeks after surgical treatment of corneal endothelial dysfunction. (a) Wound group: cornea denuded of endothelium, (b) OFHA group: endothelial scrape-wounded cornea implanted with HA carriers, and (c) OFHA+CEC group: endothelial scrape-wounded cornea implanted with HA carriers and bioengineered CEC sheet.

Fig 8 shows the morphology of CECs in rabbit eyes observed by specular microscopy. At postoperative 4 weeks, the endothelial cell counts of both the Wound and OFHA groups were not available from specular microscopic images given that no corneal endothelium was shown on denuded Descemet’s membrane. In the OFHA+CEC groups, the presence of endothelial cells on the posterior surfaces of cornea was evident. The cellular hexagonality could also be identified, suggesting intact corneal endothelial morphology. At pre-operation, the mean CEC density was 3289 ± 70 cells/mm^2^. No significant difference was noted in the cell density of OFHA+CEC groups (3216 ± 95 cells/mm^2^) compared with the values before surgery (*P* > 0.05).

**Fig 9:**
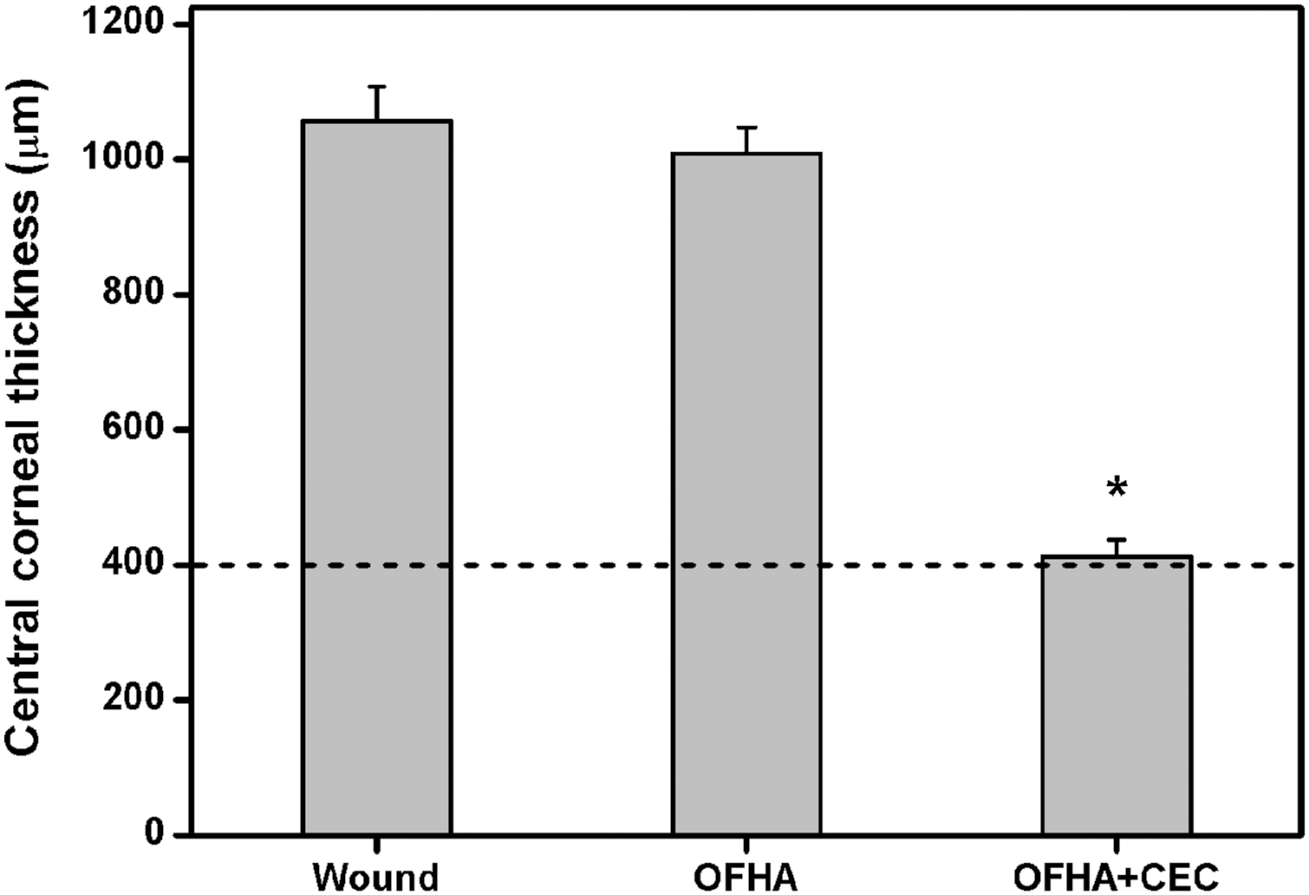
Measurements of central corneal thickness 4 weeks after surgical treatment of corneal endothelial dysfunction. Wound group: cornea denuded of endothelium, OFHA group: endothelial scrape-wounded cornea implanted with HA carriers, and OFHA+CEC group: endothelial scrape-wounded cornea implanted with HA carriers and bioengineered CEC sheet. The dash line represents the preoperative corneal thickness. An asterisk indicates statistically significant differences (*P < 0.05; *n* = 6) as compared to the Wound groups.

Fig 9 illustrates the mean CCT changes of the surgical corneas of the three groups. Mean preoperative corneal thickness was 401.3 ± 19.6 μm. An increase in CCT occurs following the removal of rabbit corneal endothelium. Therefore, in the Wound groups, the endothelial scrape-wounded corneas turned edematous at 4 weeks postoperatively. The CCT of the OFHA-implanted eyes increased to a value greater than 1000 μm. However, in the OFHA+CEC groups, the thickness value was 412.7 ± 25.0 μm, which was not significantly different compared with those before surgery (*P* > 0.05). Our data clearly demonstrate that these cell/biopolymer constructs are able to alleviate corneal edema. Transplantation of bioengineered CEC sheets using overrun-processed and freeze-dried HA carriers is a promising way to restore corneal transparency and improve vision.

The use of biomaterial carriers for cultivated CEC transplantation has attracted considerable attention. As a native extracellular matrix protein, collagen is one of the most commonly used biomaterials for corneal endothelial regenerative medicine [41]. Levis et al. have devised a strategy for ex vivo expansion and in vivo transplantation of human CECs by using plastic compressed collagen as a highly effective carrier [42]. A recent study from Yoshida et al. has reported that a transplantable artificial endothelial graft can be created by combining cultured human CECs and the biocompatible porcine-derived atelocollagen vitrigel membrane with a spherical curvature [43]. To generate transplantable human CEC layers for corneal tissue engineering, Teichmann et al. have designed a novel thermo-responsive cell carrier based on simultaneous electron beam immobilization and cross-linking of poly(vinyl methyl ether) on polymeric surfaces [44]. In this study, we pay particular attention to the development of overrun-processed and freeze-dried HA hydrogels as CEC sheet carriers.

## Conclusions

Clinically, the loss of CECs causes corneal opacity and failure and eventually leads to serious vision problems. In the present work, we first propose the use of overrun-processed and freeze-dried HA hydrogels as cell sheet carriers in corneal endothelial tissue engineering. During the fabrication of biopolymer carriers, an additional nitrogen gas injection in HA solutions is beneficial to enlarge the pore structure and prevent dense surface skin formation. Among all the groups studied, the disc samples from OFHA groups show the greatest biological stability, the highest freezable water content and glucose permeability, and the minimized adverse effects on CEC homeostasis. After intraocular delivery by OFHA carriers, the bioengineered cell sheets can reconstruct corneal endothelium and restore corneal clarity. Furthermore, the corneal edema is greatly reduced, suggesting the function of implanted cell/biopolymer constructs.
